# Outcomes of Sentinel Lymph Node Dissection Alone vs. Axillary Lymph Node Dissection in Early Stage Invasive Lobular Carcinoma: A Retrospective Study of the Surveillance, Epidemiology and End Results (SEER) Database

**DOI:** 10.1371/journal.pone.0089778

**Published:** 2014-02-25

**Authors:** Jun Wang, Elizabeth A. Mittendorf, Aysegul A. Sahin, Min Yi, Abigail Caudle, Kelly K. Hunt, Yun Wu

**Affiliations:** 1 Department of Oncology, General Hospital, Jinan Command of People's Liberation Army, Jinan, China; 2 Department of Pathology, The University of Texas MD Anderson Cancer Center, Houston, Texas, United States of America; 3 Department of Surgical Oncology, The University of Texas MD Anderson Cancer Center, Houston, Texas, United States of America; University Medical Centre Utrecht, The Netherlands

## Abstract

**Background:**

The American College of Surgeons Oncology Group (ACOSOG) Z0011 trial demonstrated no difference in local-regional recurrence (LRR), disease-specific survival (DSS) or overall survival (OS) for sentinel lymph node dissection (SLND) and completion axillary lymph node dissection (ALND) among patients undergoing breast-conserving therapy for clinical T1–T2, N0 breast cancer with 1 or 2 positive SLNs. However, Only 7% of study participants had invasive lobular carcinoma (ILC). Because ILC has a different pattern of metastases, frequently presenting as small foci requiring immunohistochemistry for detection, the applicability of ACOSOG Z0011 trial data to ILC patients is unclear.

**Study Design:**

We identified all ILC patients in the Surveillance, Epidemiology, and End Results (SEER) database (1998–2009) who met the ACOSOG Z0011 eligibility criteria. Patients were evaluated on the basis of the extent of axillary surgery (SLND alone or ALND), and the clinical outcomes of these 2 groups were compared.

**Results:**

1269 patients (393 SLND and 876 ALND) were identified from the SEER database. At a median follow-up time of 71 months, there were no differences in OS or disease-specific survival between the two groups.

**Conclusion:**

SLND alone may result in outcomes comparable to those achieved with ALND for patients with early-stage ILC who meet the ACOSOG Z0011 eligibility criteria.

## Introduction

Sentinel lymph node dissection (SLND) is the standard method of nodal staging in patients with clinically node-negative breast cancer. Until the publication of the American College of Surgeons Oncology Group (ACOSOG) Z0011 trial results, completion axillary lymph node dissection (ALND) was recommended when the sentinel lymph node (SLN) demonstrated metastatic carcinoma. The ACOSOG Z0011 randomized trial was designed to determine whether SLND alone was not inferior to completion ALND in patients with clinical T1–T2, N0 breast cancer found to have one or two positive SLNs. All patients in the Z0011 trial underwent breast conserving therapy (BCT), including lumpectomy and whole breast irradiation. The primary endpoint was overall survival (OS): at a median follow-up of 6.3 years, there was no difference in OS between the ALND and SLND arms (91.8% vs. 92.5%, respectively). Local-regional recurrence (LRR) was a secondary endpoint, and again, no differences were seen between the arms. Local recurrence rates were 3.6% in the ALND arm and 1.8% in the SLND arm, whereas ipsilateral axillary recurrences occurred in 0.5% of patients in the ALND arm and 0.9% of patients in the SLND arm [1], [2].

Invasive lobular carcinoma (ILC) is the second most common histologic type of invasive mammary carcinoma, comprising 5%–15% of all invasive breast carcinomas [3]. Practically, ILC is not treated differently from invasive ductal carcinoma (IDC); however, ILC has several unique features. Most ILCs are well differentiated and estrogen receptor (ER) positive; they tend to have a multifocal, multicentric and bilateral distribution; and they often have a dispersed growth pattern both in the breast and at metastatic sites including the axillary lymph nodes. Detection of nodal disease sometimes requires immunohistochemical staining for cytokeratin for identification. ^3^ In addition, ILC patients tend to be at risk for distant recurrence for more than 5–10 years [4].

Only 63 (7%) of the 856 patients in ACOSOG Z0011 had ILC; thus, it is unclear whether the trial's results are applicable to such patients. The objective of the current study was to determine whether the Z0011 trial's results could be safely applied to ILC patients. We used the Surveillance Epidemiology and End Results (SEER) database to identify ILC patients who met the ACOSOG Z0011 eligibility criteria. Patients were evaluated on the basis of the extent of axillary surgery (SLND alone or ALND), and the clinical outcomes of these 2 groups were compared.

## Patients and Methods

### Data acquisition and patient selection

**Figure 1:**
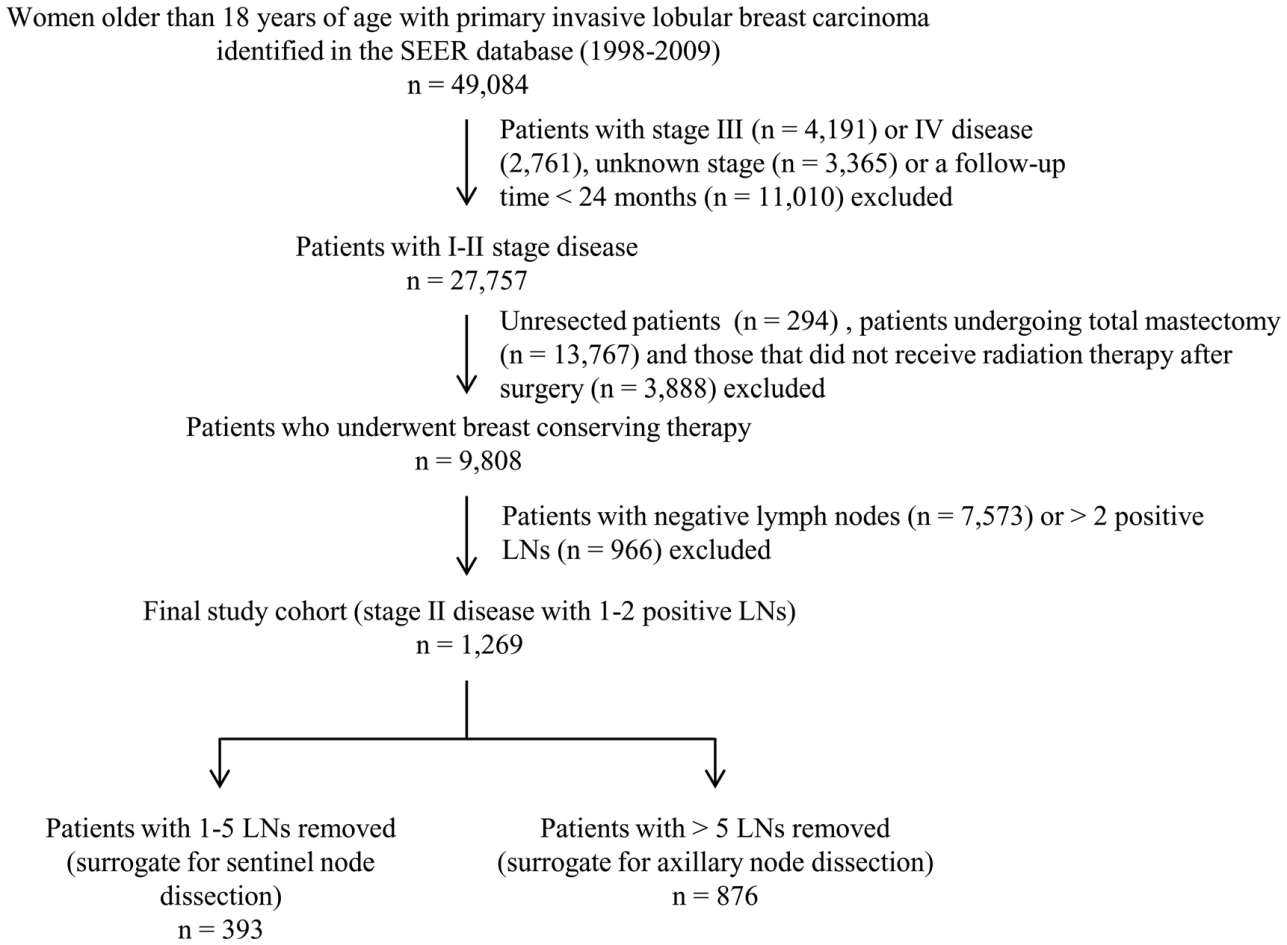
Algorithm for patient selection. The Surveillance Epidemiology and End Results 1998–2009 database was used to identify patients diagnosed with invasive lobular carcinoma (ILC). Patients were excluded if their disease stage was unknown, if they had stage III or stage IV disease, if their follow-up time was less than 24 months, if they did not undergo surgery, underwent mastectomy or did not receive radiation as a component of breast conserving therapy. Patients who underwent breast conserving therapy (BCT) who had more than 2 positive lymph nodes were also excluded. This left a final study cohort of 1,269 patients with T1–T2 ILC with 1–2 positive lymph nodes who underwent BCT.

The SEER database was used to identify 49,084 patients older than 18 years of age who had been treated for ILC from January 1998 to November 2009 using the International Classification of Diseases (ICD) code 8520/3. Patients were excluded if they had stage III (n = 4,191) or IV disease (n = 2,761), unknown stage (n = 3,365), had a follow-up duration of <24 months (n = 11,010), did not undergo surgical resection (n = 294), underwent total mastectomy (n = 13,767), did not receive post-operative radiotherapy (n = 3,888), were node negative (n = 7,573) or had 3 or more positive lymph nodes (n = 966). The remaining 1,269 ILC patients— those who had T1–T2 tumors and 1 or 2 positive lymph nodes and underwent BCT— were included in our study. The SEER database does not specify the axillary lymph node surgery performed; therefore, surrogates were used to categorize patients as having undergone SLND or ALND. Patients with 1–5 lymph nodes removed were considered to have undergone SLND alone, whereas patients with more than 5 lymph nodes removed were considered to have undergone ALND. These definitions were based on the American Joint Committee on Cancer (AJCC) definition of a standard low axillary lymph node dissection (at least 6 lymph nodes) [5]. Using these definitions, we assigned 393 patients to the SLND group and 876 patients to the ALND group (Figure 1).

The SEER database also does not provide specific information regarding LRR. Therefore, we identified patients with 2 or more registered entries after the primary surgery. If the same breast was affected, it was counted as an ipsilateral breast tumor recurrence (IBTR); if the lymph nodes were affected, it was counted as an ipsilateral regional recurrence.

### Statistical analyses

The differences in categorical variables and proportions between the SLND and ALND groups were evaluated using the χ^2^ test or Fisher's exact test as appropriate. Age and tumor size were analyzed as continuous variables, and statistical differences in the mean values were assessed using Student's t test. Disease-specific survival (DSS) and OS rates were used as primary endpoints. Survival was measured from the date of diagnosis to the date of death, the date last known to be alive, or November 30, 2009. Patients were coded as censored if they were lost to follow-up or survived beyond November 30, 2009. To determine the effects of different variables on OS and DSS, we performed a univariate survival analysis using the Kaplan-Meier method, and the significance was assessed using the log-rank test. A multivariate analysis was performed using the Cox proportional hazards model. The estimated risks for OS or DSS were calculated as hazard ratios (HRs) with 95% confidence intervals (CIs).

All tests were 2-tailed, and a *P*-value <0.05 was considered statistically significant. Statistical analyses were performed using STATA software version 11.0 (Stata Corporation, College Station, TX, USA).

## Results

### Clinicopathologic characteristics

The study population consisted of 1,269 patients with T1–T2 ILC with one or two positive lymph nodes who underwent BCT; 393 in the SLND group and 876 in the ALND group. Table 1 lists the clinicopathologic characteristics of both cohorts. The groups were well matched except that patients in the SLND group were older (median age of 63.5 years versus 60.5 years; p<.001) and more likely to have only 1 positive lymph node (88.3% vs. 69.3%; p<.001).

**Table 1:** Comparison of clinicopathologic characteristics between patients with T1–T2 ILC and 1–2 positive lymph nodes undergoing SLND alone and those undergoing ALND.

Clinicopathologic Features	SLND alone		ALND		*p* *
	*n*	%	*n*	%	
**Race**					0.24
White	360	91.6	775	88.5	
Black	19	4.8	58	6.6	
Other	14	3.6	43	4.9	
**Age (years)**					
Mean	63.5		60.5		<0.0001
Median (range)	64 (35–91)		60 (28–87)		
**Tumor size (mm)**					
Mean	18.7		19.7		0.09
Median (range)	17 (1–50)		18 (1–50)		
**T stage**					0.14
T1	266	67.7	555	63.4	
T2	127	32.3	321	36.6	
**Histologic grade**					0.16
I	97	24.7	173	19.7	
II	171	43.5	390	44.5	
III	40	10.2	104	11.9	
Unknown	85	21.6	209	23.9	
**Number of positive LNs**					<0.0001
1	347	88.3	607	69.3	
2	46	11.7	269	30.7	
**ER status**					0.17
Negative	7	1.8	28	3.2	
Positive	355	90.3	795	90.8	
Unknown	31	7.9	53	6.1	
**PR status**					0.63
Negative	66	16.8	140	16.0	
Positive	290	73.8	666	76.0	
Unknown	37	9.4	70	8.0	

Abbreviations: SLND, sentinel lymph node dissection; ALND, axillary lymph node dissection; LN, lymph node; ER, estrogen receptor; PR, progesterone receptor.

^*^Cases with unknown status were excluded from statistical analysis.

### Survival analyses

**Figure 2:**
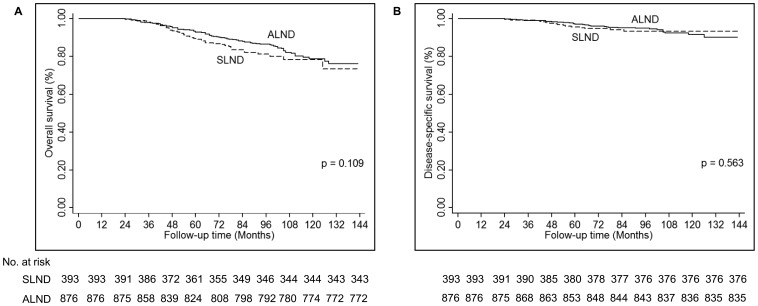
Survival outcomes for patients with T1–T2 ILC with 1–2 positive lymph nodes who underwent breast conserving therapy. No differences were identified in overall survival (A) or disease-specific survival (B) for patients who underwent sentinel lymph node dissection alone compared to those who underwent axillary lymph node dissection.

The median follow-up duration was 73 months (range 24–143 months). There were no LRRs reported in the SLND group and only 3 (0.21%) IBTR and 1 (0.07%) regional recurrence was reported in the ALND group, indicating that LRR is uncommon after BCT among these patients with T1–T2 ILC with 1–2 positive nodal metastases. There were no differences in OS or DSS between the SLND and ALND groups (Figure 2). The 5- and 10-year OS rates were 89.4% and 78.3%, respectively in the SLND group and 92.9% and 78.7% in the ALND group. The 5- and 10-year DSS rates were 95.6% and 93.3% in the SLND group and 97.0% and 91.5% in the ALND group (Table 2).

**Table 2:** Overall and disease-specific survival of patients with T1-T2 invasive lobular carcinoma and 1-2 positive lymph nodes who underwent breast conserving therapy.

Cohorts	5-year OS (95% CI)	10-year OS (95% CI)	5-year DSS (95% CI)	10-year DSS (95% CI)
**All (1-2 positive LNs)**				
SLND alone (n = 393)	89.4 (85.4–92.4)	78.3 (71.1–84.0)	95.6 (92.5–97.5)	93.3 (89.1–95.9)
ALND (n = 876)	92.9 (90.8–94.6)	78.7 (73.9–82.7)	97.0 (95.4–98.1)	91.5 (87.7–94.2)
**1 positive LNs**				
SLND alone (n = 347)	90.1 (85.8–93.2)	77.4 (68.9–83.8)	96.1 (92.9–97.9)	94.0 (89.5–96.6)
ALND (n = 607)	93.6 (91.1–95.5)	79.5 (73.7–84.1)	98.0 (96.2–98.9)	92.4 (87.5–95.4)
**2 positive LNs**				
SLND alone (n = 46)	84.3 (68.2–92.7)	81.2 (64.3–90.7)	91.8 (76.4–97.3)	88.4 (71.6–95.6)
ALND (n = 269)	91.3 (86.8–94.3)	77.1 (67.9–84.0)	94.9 (90.9–97.2)	89.7 (82.4–94.1)

Abbreviations: CI, confidence interval; OS, overall survival; DSS, disease-specific survival; LN, lymph node; SLND, sentinel lymph node dissection; ALND, axillary lymph node dissection.

**Figure 3:**
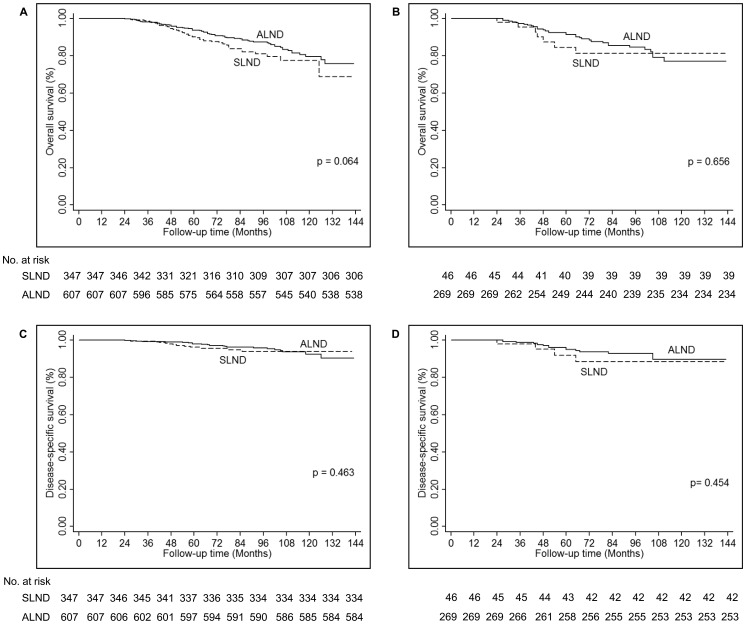
Survival outcomes based on the number of positive lymph nodes. Overall survival (A and B) and disease-specific survival (C and D) were not different among patients who underwent SLND alone and those who underwent ALND for patients with one positive lymph node (A and C) or two positive lymph nodes (B and D).

Because a higher percentage of patients in the SLND alone group had only 1 positive lymph node, we next compared the OS and DSS rates between the 2 groups, evaluating patients with the same number of positive lymph nodes. Among patients with 1 positive lymph node, there were no differences in OS (Figure 3A) or DSS (Figure 3C) comparing patients that underwent SLND alone to those that underwent ALND. Similarly, there were no differences in either survival endpoint for patients with 2 positive lymph nodes when comparing patients that underwent SLND alone to those that underwent ALND (Figure 3B and 3D).

Because the ACOSOG Z0011 trial accrual was between May 1999 and November 2004, we repeated the analyses from 1999 to 2004. No statistically significant difference was found in OS or DSS between patients who underwent SLND only and those who underwent ALND (data not shown).

Although the ACOSOG Z0011 trial was written to include only patients with one or two positive SLNs, due to the fact that some patients underwent intraoperative randomization, a small percentage (3.7%) of patients in the SLND alone arm had 3 or more positive lymph nodes identified. ^1^ We therefore repeated our analyses looking at patients with one, two or three positive lymph nodes. Again, there were no differences in OS or DSS when comparing patients with up to three positive lymph nodes who underwent SLND alone versus ALND (data not shown).

### Prognostic factors associated with OS and DSS

In addition to the extent of axillary surgery performed, we evaluated other clinicopathologic factors, including patient race and age, tumor sizeand histologic grade, the number of postive lymph nodes, and ER and PR status to determine their effects on OS and DSS. On univariate analysis (Table 3), older age (>50 years) and large tumor size (T2 vs T1) were associated with poorer OS. Larger tumor size (T2 vs T1) was the only statistically significant factor associated with reduced DSS. The extent of axillary surgery was not significantly associated with either OS or DSS. In a multivariate analysis of all the factors (Table 4), age >50 years and large tumor size (T2 vs T1) were identified as independent prognositic factors associated with reduced OS. Large tumor size (T2 vs T1) was the only independent prognostic factors associated with poorer DSS.

**Table 3:** Univariate analysis of prognostic factors for disease-specific survival and overall survival in patients with T1-T2 invasive lobular carcinoma and 1-2 positive lymph nodes who underwent breast conserving therapy.

Variable	DSS		OS	
	HR (95% CI)	*p*	HR (95% CI)	*p*
**Race**				
White	Reference		Reference	
Black	0.30 (0.42–2.20)	0.29	0.78 (0.36–1.66)	0.52
Other	1.21 (0.38–3.87)	0.75	0.59 (0.22–1.60)	0.30
**Age (years)**				
≤50	Reference		Reference	
>50	1.03 (0.53–1.99)	0.09	2.34 (1.37–3.99)	0.00
**T stage**				
T1	Reference		Reference	
T2	2.29 (1.36–3.88)	0.00	1.38 (1.00–1.90)	0.05
**Number of positive LNs**				
1	Reference		Reference	
2	1.63 (0.94–2.82)	0.08	1.10 (0.77–1.57)	0.59
**Histologic Grade**				
I	Reference		Reference	
II	1.47 (0.69–3.13)	0.32	1.09 (0.69–1.73)	0.71
III	1.96 (0.79–4.85)	0.12	1.70 (0.98–2.94)	0.06
**ER status**				
Negative	Reference		Reference	
Positive	0.48 (0.20–1.11)	0.09	0.66 (0.39–1.10)	0.11
**PR status**				
Negative	Reference		Reference	
Positive	0.76 (0.46–1.27)	0.30	0.78 (0.57–1.06)	0.11
**Axillary surgery**				
SLND alone	Reference		Reference	
ALND	0.85 (0.48–1.50)	0.57	0.76 (0.54–1.06)	0.10

Abbreviations: DSS, disease-specific survival; OS, overall survival; LN, lymph node; ER, estrogen receptor; PR, progesterone receptor; SLND, sentinel lymph node dissection; ALND, axillary lymph node dissection.

**Table 4:** Multivariate analysis of prognostic factors for disease-specific survival and overall survival in patients with T1–T2 invasive lobular carcinoma and 1-2 positive lymph nodes who underwent BCT.

Variable	DSS		OS	
	HR (95% CI)	*p*	HR (95% CI)	*p*
**Race**				
White	Reference		Reference	
Black	0.30 (0.04–2.15)	0.23	0.79 (0.37–1.69)	0.54
Other	1.27 (0.39–4.11)	0.69	0.65 (0.24–1.75)	0.39
**Age (years)**				
≤50	Reference		Reference	
>50	1.00 (0.52–1.95)	1.00	2.25 (1.32–3.86)	0.00
**T stage**				
T1	Reference		Reference	
T2	2.24 (1.32–3.82)	0.00	1.40 (1.01–1.94)	0.04
**Number of positive LNs**				
1	Reference		Reference	
2	1.66 (0.95–2.92)	0.08	1.19 (0.82–1.70)	0.39
**Histologic grade**				
I	Reference		Reference	
II	1.44 (0.67–3.08)	0.35	1.11 (0.70–1.77)	0.67
III	1.92 (0.77–4.74)	0.16	1.71 (0.98–2.96)	0.06
**ER status**				
Negative	Reference		Reference	
Positive	0.55 (0.21–1.45)	0.23	0.77 (0.42–1.39)	0.39
**PR status**				
Negative	Reference		Reference	
Positive	0.86 (0.46–1.58)	0.62	0.90 (0.63–1.29)	0.56
**Axillary surgery**				
SLND alone	Reference		Reference	
ALND	0.71 (0.40–1.28)	0.26	0.75 (0.53–1.06)	0.11

Abbreviations: DSS, disease-specific survival; OS, overall survival; LN, lymph node; ER, estrogen receptor; PR, progesterone receptor; SLND, sentinel lymph node dissection; ALND, axillary lymph node dissection.

## Discussion

Breast conserving therapy including lumpectomy and whole breast irradiation has become widely used in the treatment of early-stage breast cancers. Although there was initial concern about treating ILC with BCT because of its tendency to be multifocal and multicentric, available data confirm that BCT is as effective for ILC as for IDC. There is no difference in the reported LRR or OS rates between ILC and IDC after BCT [6], [7]. The results of the ACOSOG Z0011 trial will likely lead to further reductions in the extent of surgery for early-stage invasive breast cancers; in particular, ALND can be omitted in patients undergoing BCT for clinical T1–T2, N0 breast cancer found to have 1 or 2 positive SLNs. Because only 7% of participants in the ACOSOG Z0011 trial had ILC, we used SEER data to confirm that the Z0011 trial results are applicable to ILC patients.

The primary endpoint of the ACOSOG Z0011 study was OS; therefore, it is important that the current study revealed no differences in OS between patients with ILC who underwent SLND and those who underwent ALND. This is in part due to the fact that these patients' tumors had very favorable biological characteristics: most were ER+ and HER2 negative, and thus likely of the molecular luminal A subtype. These patients are generally treated with endocrine therapy for five years. Although data regarding systemic therapy is not available in the SEER database, given the years included in this analysis (1998–2009), most patients were likely treated with adjuvant endocrine therapy.

A secondary endpoint of the ACOSOG Z0011 study was LRR. In that trial, after a median follow-up of 6.3 years, the local recurrence rates were1.8% and 3.6% in the SLND and ALND arms, respective [2]. The regional recurrence rates were 0.9% and 0.5% in the SLND and ALND arms. In the current study, we found that after a median follow-up duration of 73 months, no LRR was reported in the SLND group, and only 3 IBTRs (0.34%) and 1 (0.11%) regional recurrence was reported in the ALND group. Of note, the SEER database does not specifically report LRR data; therefore, we used surrogates. Specifically, we identified patients with 2 or more record entries after the primary surgery. If the same breast was affected it was counted as an IBTR and if lymph nodes were affected, it was counted as an ipsilateral regional recurrence. This likely underestimates the LRR risk. Despite this, the LRR rates in this population may be low regardless of the extent of axillary surgery performed, partly because of the overall favorable biological characteristics of ILC, which is predominantly ER+, luminal A type. In the current study, 90.6% of patients had ER+ tumors versus 74.8% in the ACOSOG Z0011 study (with ER status unknown in 9.5%) [1]. Our results are consistent with those of a recent study by Arvold et al that found a LRR rate of 0.8% in patients with luminal A breast cancer approximated as hormone receptor (HR)-positive, HER2-negative, grade 1–2 after BCT compared to 2.3% in luminal B (HR+, HER2−, grade 3), 1.1% in HER2+ luminal B (HR+, HER2+), 10.8% in HER2-enriched (HR−, HER2+) and 6.7% in basal (HR−, HER2−) cancers [8].

This study has several limitations. First, in contrast to the ACOSOG Z0011 study which was a randomized trial, our study was a retrospective review of a large, population-based database. There may have been bias with respect to which patients underwent SLND alone in that surgeons selected patients for this limited axillary surgery on the basis of perceived favorable biologic characteristics. This is consistent with previously published data from the SEER database showing a trend towards omitting ALND in selected patients, specifically, older women with low-grade, ER-positive tumors [9]. Similarly, a review of National Cancer Data Base (NCDB) data revealed a trend towards omitting ALND in patients with micrometastases in the SLN [10]. A patient selection bias was also present in the ACOSOG Z0011 trial. In a study evaluating factors influencing participation in the trial, Leitch et al. reported that 69% of SLN positive patients who were eligible to participate but did not enroll and underwent an ALND instead. This bias contributed to the overall very favorable characteristics of patients enrolled in the ACOSOG Z0011 trial [11]. Although there were some differences between the patients reported in the current study and those in the ACOSOG Z0011 trial—slightly fewer T1 tumors (64.7% vs. 68.6%) and more ER-positive tumors (90.6% vs. 74.8%)—both studies included patients with very favorable biologic characteristics.

A second limitation is that the SEER database does not specify whether a patient underwent SLND alone or ALND. We therefore used surrogates; patients with 5 or fewer lymph nodes removed were categorized as having undergone SLND alone, whereas patients with >5 lymph nodes removed were categorized as having undergone ALND. There may be a concern that using 5 or fewer nodes removed as a surrogate for SLND may potentially lead to poorer prognosis due to insufficient axillary node dissection. In contrast, using 6 and more nodes removed as a surrogate for ALND may potentially lead to better prognosis. Nevertheless, the analyses from the SEER database showed no difference in OS or DSS in the SLND and ALND groups using the above surrogates, despite the potential survival difference caused by arbitrary subgrouping, indicating that it may be safe to perform SLND in early stage (clinical T1 and T2, N0) ILC. These surrogates are consistent with the AJCC definition of an ALND and were used in the above referenced study of the National Cancer Data Base dataset.

A third limitation is that the SEER database does not provide specific data on the use of radiation therapy. An important aspect of the ACOSOG Z0011 trial is that all patients underwent opposing tangential field whole breast irradiation. With these tangents, 51% of level I and 26% of level II axillary lymph nodes receive 95% of the prescribed dose [12], radiation may have contributed to the favorable local-regional control demonstrated in the ACOSOG Z0011 trial. Thus, the ACOSOG Z0011 data are only applicable to patients undergoing BCT with whole breast irradiation and should not be applied to those underwent accelerated partial-breast irradiation (APBI) or radiation administered in the prone position [13]. With respect to APBI, the American Society of Radiation Oncology (ASTRO) consensus statement recommends that patients with ILC be included in a “cautionary” group with respect to considering APBI based on randomized clinical trial data showing a higher IBTR risk in patients with ILC compared to IDC when treated with APBI [14]–[16]. The SEER database does not provide specific information regarding the modality by which radiation was administered; however, given the years of the study, the relatively newness of accelerated partial-breast irradiation, and the identification of ILC patients as a cautionary group by by the American Society of Radiation Oncology (ASTRO), we believe that most patients in the current study would have undergone standard whole breast irradiation administered in the supine position.

Finally, our study population was restricted to patients with pathologic T1 and T2 tumors, whereas the ACOSOG Z0011 study enrolled patients with clinical T1 and T2 tumors. Although all patients in the ACOSOG Z0011 trial had clinical T1 or T2 tumors, the median tumor size and range indicated a percentage of patients had pathologic T3 disease (the median tumor size in the ALND arm was 1.7 cm; range 0.4–7.0) [1]. ILC has a diffuse growth pattern and rarely forms a mass lesion; therefore, clinical measurement of the tumor size by palpation or radiographic evaluation may underestimate the disease extent. Caution is advised regarding application of the ACOSOG Z0011 data to patients with clinical T1 or T2 ILC that has larger extent identified on pathologic evaluation.

In conclusion, the results of current study demonstrate that among patients with T1–T2 ILC and low volume nodal metastasis, there is no difference in LRR, DSS or OS for patients undergoing SLND alone compared to those undergoing ALND. These findings suggest that, consistent with the findings from the ACOSOG Z0011 trial, SLND alone, without completion ALND may result in comparable outcomes for patients with early-stage ILC. However, given the limitations of this retrospective cohort study, further investigation is warranted to validate these findings.
